# Geranyl hydroquinone alleviates rheumatoid arthritis-associated pain by suppressing neutrophil accumulation, N1 polarization and ROS production in mice

**DOI:** 10.1016/j.redox.2025.103603

**Published:** 2025-03-18

**Authors:** Sen Huang, Yuxin Xie, Zhaochun Zhan, Fengdong Liu, Peiyang Liu, Fei Xu, Tingting Xu, Zhenning Fang, Zhiqiang Chen, Qingjian Han, Ligang Jie, Rougang Xie, Hongfei Zhang, Shiyuan Xu, Yiwen Zhang, Kai Mo, Xin Luo

**Affiliations:** a Department of Anesthesiology, Zhujiang Hospital, Southern Medical University, Guangzhou, 510280, China; b Key Laboratory of Mental Health of the Ministry of Education, Guangdong-Hong Kong-Macao Greater Bay Area Center for Brain Science and Brain-Inspired Intelligence, Guangdong-Hong Kong Joint Laboratory for Psychiatric Disorders, Guangdong Province Key Laboratory of Psychiatric Disorders, Guangdong Basic Research Center of Excellence for Integrated Traditional and Western Medicine for Qingzhi Diseases, Department of Neurobiology, School of Basic Medical Sciences, Southern Medical University, Guangzhou, 510515, China; c Department of Anesthesiology, Shunde Hospital, Southern Medical University, Foshan, 528300, China; d State Key Laboratory of Medical Neurobiology and MOE Frontiers Center for Brain Science, Institutes of Brain Science, Fudan University, Shanghai, 200032, China; e Department of Neurobiology, School of Basic Medicine, Fourth Military Medical University, Xi'an, 710032, China; f Department of Rheumatology and Clinical Immunology, Zhujiang Hospital, Southern Medical University, Guangzhou, 510280, China; g Institute of Perioperative Medicine and Organ Protection, Zhujiang Hospital, Southern Medical University, Guangzhou, 510280, China

**Keywords:** Rheumatoid arthritis, Pain, Neutrophils, Reactive oxygen species, Microsomal glutathione S-Transferase 3

## Abstract

Pain hypersensitivity is a hallmark of rheumatoid arthritis (RA); however, the underlying mechanisms and effective therapies remain largely undefined. Emerging studies suggest that neutrophils play a significant role in the pathology of RA, yet their involvement in RA-associated pain is still unclear. The present study investigates whether neutrophil activity contributes to pain pathogenesis in RA. Our flow cytometry analysis reveals that the accumulation and N1 polarization (indicated by the ratio of CD45^+^CD66b^+^CD95^+^ subset) of neutrophils occur in synovial fluid samples from RA patients, positively correlating with pain scores. In the collagen-induced rheumatoid arthritis (CIA) model, mice demonstrate neutrophil accumulation, N1 polarization (indicated by the ratio of CD45^+^Ly-6G^+^CD95^+^ subset), and reactive oxygen species (ROS) production in affected paw tissues. Geranyl hydroquinone (GHQ), a natural meroterpenoid with antioxidative properties, reverses N1 polarization and ROS production in synovial neutrophils from RA patients *in vitro*. Moreover, a 10-day oral administration of GHQ alleviates pain hypersensitivity and reduces neutrophil accumulation, N1 polarization, and ROS production in CIA mice. Notably, GHQ treatment reverses TNF-α-evoked ROS production in neutrophils *in vitro* through downregulating gene expression associated with the ROS pathway. Further, liquid chromatography-tandem mass spectrometry and biochemical analyses indicate that GHQ binds to microsomal glutathione S-transferase 3 (MGST3) in neutrophils. *In vitro* and *in vivo* evidence demonstrates that the RA-specific analgesic and antioxidative effects of GHQ require MGST3. Lastly, GHQ administration exhibits superior therapeutic effects compared to methotrexate, a first-line disease-modifying antirheumatic drug, in CIA mice. Collectively, our findings indicate that neutrophil accumulation, N1 polarization and ROS production contribute to RA-associated pain, suggesting that targeting these pathways, such as with GHQ, could be a viable strategy for RA treatment.

## Highlights

• Neutrophil accumulation and N1 polarization promote rheumatoid arthritis pain.
• Neutrophil-derived reactive oxygen species promote rheumatoid arthritis pain.
• MGST3 suppresses neutrophil N1 polarization and ROS production.
• Geranyl hydroquinone relieves RA pain via suppressing neutrophil disorder.

## Graphical abstract

Huang et al. found that the accumulation, N1 polarization and ROS production of synovial neutrophils promote the pathogenesis of RA-associated pain. GHQ, a meroterpenoid compound, exerts RA-specific analgesic and anti-oxidative effects. Notably, GHQ requires MGST3 to inhibit ROS production, thereby reversing neutrophil N1 polarization under RA condition.

**Figure:**
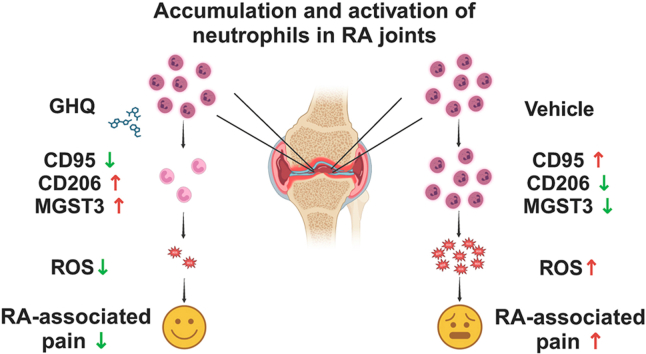

## 1 Background

Rheumatoid arthritis (RA) is one of the most common autoimmune diseases, which is characterized by symmetric polyarticular pain symptoms and inflammation. As RA-associated pain can impair physical and social functioning individuals, achieving remission of pain symptoms is a primary medical treatment goal for RA patients [1,2]. However, the underlying pathology of RA-associated pain remains poorly understood, which limits the development of effective, RA-specific analgesic drugs. Indeed, current treatments, such as disease-modifying anti-rheumatic drugs (DMARDs), including non-biologic agents (e.g. methotrexate) and biologic agents (e.g. anti-TNF-α monoclonal antibodies), often fail to achieve full remission in RA patients or their effects on RA-associated pain are not well examined [[3], [4], [5]]. Furthermore, the effectiveness of these treatments may be restricted by potential adverse events, such as cytotoxicity and insufficient long-term efficacy [6].

Neutrophils, the predominant type of myeloid leukocyte, are among the first responders of the innate immune system in various disease conditions [7]. By interacting with signaling molecules released in the microenvironment, their functional phenotype can be altered through a process known as 'polarization'. Human neutrophils can polarize into two major functional states: the pro-inflammatory N1 state (indicated by the ratio of CD45^+^CD66b^+^CD95^+^ subset) and the anti-inflammatory N2 state (indicated by the ratio of CD45^+^CD66b^+^CD206^+^ subset) [8]. Increasing evidence shows that neutrophils accumulate and become activated in RA joints, where they release cytokines and chemokines, promote protein citrullination, and produce reactive oxygen species (ROS), all of which contribute to RA pathology [9,10]. Notably, our previous studies have underscored the importance of neuron-immune interactions as essential mechanisms underlying chronic pain pathology [[11], [12], [13], [14], [15]]. This suggests that synovial neutrophil may activate peripheral sensory neurons and initiate pain processing in the context of RA, which needs further investigation.

In this study, we aimed to investigate the role of neutrophil accumulation and activation in RA-associated pain. To explore this, we examined the relationship between neutrophil polarization in synovial fluid and pain scores in RA patients, and further tested this association in a mouse model of collagen-induced arthritis (CIA). Meroterpenoids are a class of natural products derived from terpenoid pathways that exert a wide range of bioactivities, including anti-inflammatory and anti-cancer effects [16]. Geranyl hydroquinone (GHQ, CAS: 10457-66-6) is a natural meroterpenoid compound with antioxidative actions [17,18]. However, it remains unclear whether GHQ alleviates RA-associated pain. To investigate it, we have applied multiple *in vivo* and *in vitro* experiments. Our results demonstrate that CIA mice exhibit pain hypersensitivity, neutrophil N1 polarization, and ROS production, all of which are reversed by GHQ treatment. Notably, GHQ binds to microsomal glutathione S-transferase 3 (MGST3, UniProt ID: O14880) in neutrophils to exert RA-specific analgesic and antioxidative effects. Together, our findings provide mechanistic insights into the pathology of RA-associated pain and may contribute to drug development for RA-specific pain management.

## 2 Materials and methods

### 2.1 Study cohort

Patients diagnosed with rheumatoid arthritis (RA) were evaluated following the 2010 American College of Rheumatology/European League Against Rheumatism (ACR/EULAR) classification criteria. The inclusion criteria for RA patients included: (1) the age was ≥18 years and the disease duration was ≥ 6 weeks, irrespective of sex; (2) Swelling in at least one joint and tenderness in at least three joints. The exclusion criteria consisted of: (1) individuals with known autoimmune diseases other than RA; (2) a history of severe chronic infection or current infection; (3) a diagnosis of cancer; and (4) pregnancy and lactation. In the osteoarthritis (OA) control group, participants had to meet the following inclusion criteria: (1) a clinical diagnosis of osteoarthritis in accordance with the Chinese Osteoarthritis Diagnosis and Treatment Guidelines (2021 edition); and (2) the age was ≥18 years, irrespective of sex. The exclusion criteria were: (1) any known autoimmune disease; (2) a history of severe chronic infection or current infection; (3) a diagnosis of cancer; and (4) pregnancy and lactation. Informed consent was obtained from all patients, which was approved by the Medical Ethics Committee of Zhujiang Hospital, Southern Medical University (approval number: 2024-KY-054-01), and registered with the National Medical Research Registration Information System (registration number: MR-44-24-015692).

### 2.2 Patient samples collection

Visual Analog Scale (VAS) scores were collected from patients diagnosed with rheumatoid arthritis and those with osteoarthritis (control) during a pre-operative visit on the day preceding surgery. Blood and joint fluid samples were obtained from patients before arthroscopy surgery, following the administration of anesthesia. All samples were transported under frozen conditions. The characteristics of the participants who provided joint fluid and synovial samples, along with their VAS scores, are presented in Table S1.

### 2.3 Animals

C57BL/6 male mice aged 8–16 weeks were obtained from Zhaoqing Ruisi Yuan Biotech Co., Ltd. (Guangzhou, China). C57BL/6Smoc-*Trpv1*^*em1(Myc-IRES-Cre)Smoc*^; C57BL/6J-*Gt(ROSA)26Sor*^*tm9(CAG-tdTomato)Hze*^/J (*Trpv1*^*CRE*^*/tdTomato*) transgenic mice were generated in our laboratory. All animals were housed under controlled conditions (12-h light/dark cycle, humidity 44 %–65 %) for an acclimation period of one week. All procedures involving experimental animals adhered to institutional and national guidelines regarding animal care and use. Animal welfare and all experimental protocols were approved by the Animal Ethics Committee of Southern Medical University (Approval No. SMUL202403011).

### 2.4 Mouse model of collagen-induced arthritis (CIA)

The CIA model was established in C57BL/6 mice as described in previous studies [19]. Briefly, the mice were immunized once intradermally with 200 μg of native chicken type II collagen (Chondrex) emulsified in an equal volume of complete Freund's adjuvant (Chondrex). The severity of arthritis was assessed and scored in a double-blind manner: 0: normal; 1: mild swelling and/or erythema of one digit; 2: moderate swelling and erythema extending from the ankle to the midfoot (tarsals); 3: severe swelling from the ankle to the metatarsophalangeal joints; 4: complete swelling and erythema involving the ankle, foot, and digits, resulting in deformity and/or ankylosis. The total clinical score (0–16) was the sum of the scores from all four limbs.

### 2.5 Behavioral tests

Von Frey Test: A series of calibrated von Frey filaments with logarithmically increasing stiffness (0.02–2.56 g; Stoelting) were applied to the plantar surface of the hindpaw, and the paw withdrawal threshold (PWT) was determined using the up-down method [20]. Hargreaves Test: Thermal hyperalgesia was measured using the Hargreaves radiant heat device (IITC Life Science). Acetone Test: Acetone (20 μL) was gently applied to the plantar surface of the hindpaw using pipette tips, and the animal's response to the acetone was scored [21]. Thermal Gradient Assay: A linear temperature gradient (5–56 °C) was established on a metal floor plate (Bioseb) [22]. All the aforementioned experiments were conducted in a double-blind manner.

### 2.6 Reagents

A list of reagents for this study is showed in Table S2.

### 2.7 Drug administration

In *in vivo* experiments, GHQ or methotrexate was administered via intragastric gavage in mice. Drugs or cells (1 × 10^5^) were delivered to the plantar surface of the hind paws through intraplantar (I.PL.) injection using a 29G needle.

### 2.8 Cell line culture

The human neutrophil cell line HL-60 (BeNa Culture Collection, Henan, China) was utilized in this study. Cells were cultured in DMEM medium supplemented with streptomycin (100 mg/mL), penicillin (100 U/mL), and 10 % fetal bovine serum (FBS) at 37 °C and 5 % CO_2_. DMSO (1.3 %) was applied *in vitro* to differentiate HL-60 cells into their functional state, referred to as dHL-60 cells. These dHL-60 cells were challenged with recombinant human TNF-α (40 ng/mL) for 6 h, during which GHQ and/or other reagents were co-administered. To knock down *MGST3*, dHL-60 cells were transfected with 2.5 μL/mL Lipofectamine® 2000 (Thermo Fisher Scientific, Waltham, USA) and 20 nM MGST3 siRNA (MCE, USA). After 6 h, the cultures were replenished with DMEM medium supplemented with 10 % FBS for an additional 48 h.

### 2.9 Primary synovial neutrophils culture

Primary synovial cells were collected from RA patients as described previously. After centrifugation, these cells were cultured in DMEM supplemented with 10 % FBS at 37 °C and 5 % CO_2_.

### 2.10 RNA-seq and data analysis

The transcriptome of dHL-60 cells from different experimental groups was analyzed using RNA-seq technology (Sequmed, Guangzhou, China). Gene Ontology (GO) functional enrichment and Kyoto Encyclopedia of Genes and Genomes (KEGG) enrichment analyses were conducted on the set of significantly differentially expressed genes, applying a threshold of padj <0.05 to determine significant enrichment. Additionally, GSEA_GO analysis and GSEA_KEGG analysis were performed on the set of targeted genes.

### 2.11 LC-MS/MS-based proteomics analysis

dHL-60 cells were incubated with Biotin-GHQ (40 μM) or Biotin (40 μM) for 6 h. Protein samples were extracted from these cells and incubated with 50 μL of streptavidin beads at 4 °C for 4 h. The streptavidin bead-enriched proteins were separated by SDS-PAGE and silver-stained for subsequent mass spectrometry analysis using the ThermoFisher Q Exactive system (ThermoFisher, USA). The original raw spectrum files were processed and analyzed with PEAKS Studio 8.5 software (Bioinformatics Solutions Inc., Waterloo, Canada). The human protein database was downloaded from UniProt. KEGG analysis was conducted on the screened potential binding targets of GHQ.

### 2.12 Biochemical assays

To determine the cytotoxicity of GHQ *in vivo*, activities of plasma aspartate aminotransferase (AST), alanine aminotransferase (ALT), blood urea nitrogen (BUN) and creatinine (CRE) in blood samples were measured using a biochemical analyzer (Toshiba TBA-40FR, Japan).

### 2.13 Immunohistochemical (IHC) assay

IHC was performed on deeply anesthetized animals as previously described [23]. These mice were perfused through the heart with PBS, followed by perfusion with 4 % paraformaldehyde. The hind paws and L3-L5 dorsal root ganglion DRG tissues were harvested and post-fixed. Data were collected using a Nikon confocal microscope (AX/AX R with NSPARC) and analyzed with ImageJ software (FIJI, National Institutes of Health, United States).

### 2.14 Flow cytometry (FC)

Hindpaw and blood samples were collected from sacrificed mice for FC [24]. Hindpaw samples were minced into small pieces and then digested with collagenase IV in RPMI 1640 medium at 37 °C for 1.5 h, followed by filtration through a 70 μm cell strainer. Blood samples were placed in heparinized centrifuge tubes and treated with red blood cell lysis buffer. Cells from these samples were stained and identified as follows: macrophages (CD45^+^CD11b^+^F4/80^+^), T cells (CD45^+^CD3^+^), B cells (CD45^+^CD19^+^), dendritic cells (DCs) (CD45^+^CD11b^+^CD11c^+^), and neutrophils (CD45^+^CD11b^+^Ly-6G^+^). The functional states of neutrophils were categorized as N1 neutrophils (CD45^+^CD11b^+^Ly-6G^+^CD95^+^) and N2 neutrophils (CD45^+^CD11b^+^Ly-6G^+^CD206^+^). Forward and side scatter (FSC/SSC) were utilized to eliminate debris and doublets. Single-cell suspensions were prepared from the synovial fluid and blood samples of RA patients. Neutrophils were stained with anti-CD45, anti-CD11b, and anti-CD66b antibodies. N1 and N2 states were labeled with anti-CD95 and anti-CD206 antibodies, respectively. All flow cytometry antibodies were purchased from BioLegend (USA). To determine intracellular reactive oxygen species (ROS) levels, single-cell suspensions from RA patients, CIA animals, or cell lines were incubated with H2DCFDA (10 μM) for 15 min. FC analysis of these samples was conducted using a BD LSRFortessa X-20 flow cytometer (BD Biosciences, San Jose, California, USA) and analyzed using FlowJo software.

### 2.15 Molecular docking

The crystal structure of MGST3 was obtained from the AlphaFold DB (UniProt ID: O14880). The energy of GHQ was minimized to a local minimum using the MMF94X force field. The AutoDock Tools version 1.5.7 package was employed to generate the docking input files and analyze the docking results, which were then visualized using PyMOL software.

### 2.16 Whole-cell patch clamp recordings

L3 and L5 DRG tissues were collected from deeply anesthetized *Trpv1*^CRE^/*Tdtomato* mice for electrophysiological testing [25]. Patch pipettes (5–8 MΩ) were pulled from borosilicate glass capillaries using a P-97 puller. A micromanipulation system was employed to align the glass electrode tip with the neuron of interest. A gigaohm seal was achieved by applying positive pressure followed by negative suction, and then further negative pressure was applied to breach the membrane. Electrical signals were recorded with an EPC-10 amplifier (HEKA Elektronik, Germany), and data were acquired and analyzed using PatchMaster software.

### 2.17 Q-PCR

Total mRNA from dHL-60 cells was extracted using the Trizol method [26]. cDNA library was constructed using 1 μg of total mRNA. The sequences of the primers for q-PCR are presented in Table S3. The amplification conditions were as follows: 95 °C for 30s, 95 °C for 5s, and 60 °C for 34s, for a total of 45 cycles. The data were analyzed using the _ΔΔ_CT method, and *β-ACTIN* serving as the internal reference gene.

### 2.18 Western blotting

Proteins were extracted using IP buffer containing a protease inhibitor mixture, separated by SDS-PAGE, and subsequently transferred to PVDF membranes. Protein levels were semi-quantified using ImageJ and normalized to the corresponding controls [27].

### 2.19 Immunoelectron microscopy

dHL-60 cells were treated with either Biotin-GHQ or vehicle for 6 h and then fixed using an electron microscopy fixative at 4 °C for 30 min. The cells were dehydrated and embedded in 812 embedding medium. Rabbit anti-MGST3 was used to label MGST3, followed by staining with colloidal gold-labeled goat anti-rabbit IgG. Biotin-GHQ was directly labeled with streptavidin-gold. Data were acquired and analyzed using a transmission electron microscope (Hitachi HT7800).

### 2.20 Chemical analysis of GHQ

High performance liquid chromatography (HPLC), high-resolution electrospray ionization mass spectrometry (HR-ESI-MS) and nuclear magnetic resonance (NMR) spectroscopy were employed to determine the structure of geranyl hydroquinone (GHQ).

### 2.21 Statistical analysis

All data are presented as mean ± SEM. Statistical analysis was performed using GraphPad Prism 9. Data normality was assessed using the Kolmogorov-Smirnov test. For normally distributed data, one-way ANOVA, two-way ANOVA, ordinary or repeated measures (RM) ANOVA, followed by Tukey's post hoc test, or unpaired/paired two-tailed Student's *t*-test were used. If data were not normally distributed, the non-parametric Mann-Whitney *U* test was applied. A p-value of <0.05 was considered statistically significant. Correlation analysis was conducted using Pearson's correlation analysis, following confirmation of normality with the Kolmogorov-Smirnov test. Detailed statistics for each experiment are summarized, which is available upon request.

## 3 Results

### 3.1 Neutrophils are recruited and polarized to N1 state in the synovial fluid of RA patients

**Fig. 1:**
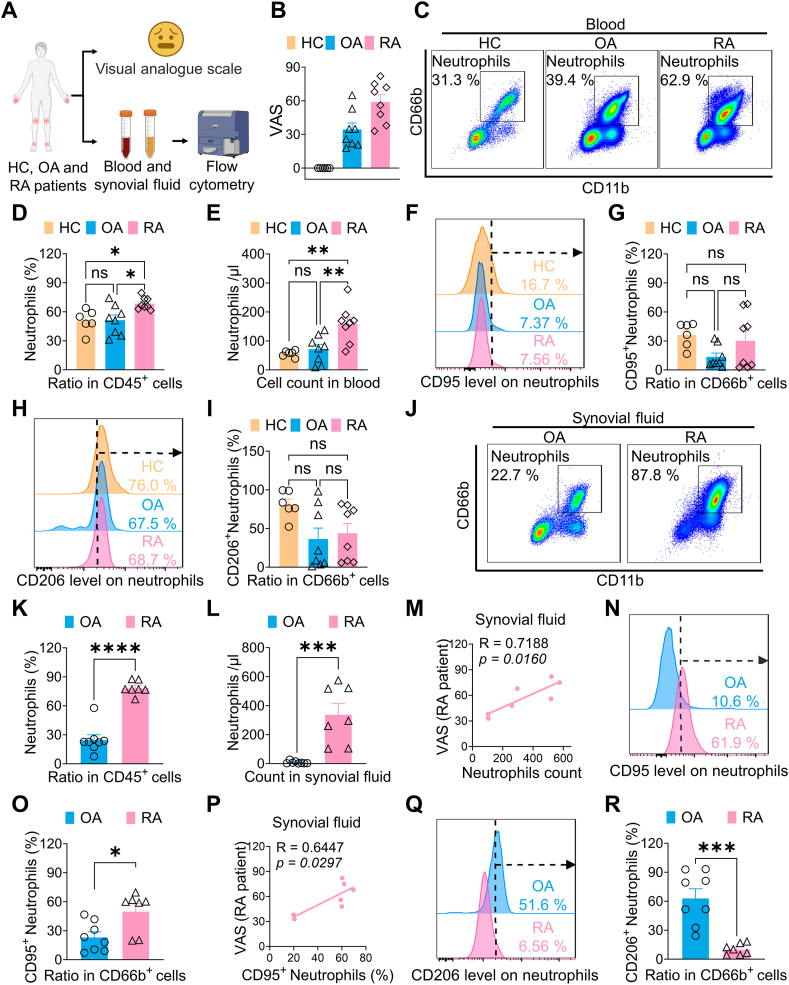
Accumulation and activation of synovial neutrophils occur in RA (A) Diagram of the research protocol using patient samples. (B) The VAS pain scores for RA and OA patients as well as healthy controls. (C) Representative flow cytometry images of neutrophils in blood samples. (D, E) The ratio and count of neutrophils in circulating leukocytes increase in RA patients compared to OA patients or healthy controls. (F–I) Expression levels of CD95 (an N1 marker) and CD206 (an N2 marker) on circulating neutrophils from RA and OA patients, and healthy controls. (J) Representative flow cytometry images of neutrophils in synovial fluid samples. (K, L) The ratio and count of neutrophils in synovial leukocytes increase in RA patients compared to OA patients. (M) Accumulation of synovial neutrophils correlates positively with the pain scores of RA patients. (N–R) Expression levels of CD95 (an N1 marker) and CD206 (an N2 marker) on synovial neutrophils from RA and OA patients. N1 polarization of synovial neutrophils correlates positively with the pain scores of RA patients. Data are mean ± SEM. ∗p < 0.05, ∗∗p < 0.01, and ∗∗∗p < 0.001, one-way ANOVA assay followed by Tukey's post hoc test (D, E, G, I), student *t*-test (K, L, O, R), Pearson correlation analysis (M, P).

Accumulation and activation of neutrophils are well-established factors in the pathology of RA [9,10]. However, it remains unclear whether these neutrophil activities are associated with pain symptoms in the context of RA. To investigate this, we collected blood and synovial fluid samples from patients with RA and osteoarthritis (OA), as well as blood samples from healthy controls. Using flow cytometry, we measured the population and polarization of neutrophils in these samples (Fig. 1A). VAS pain scoring data indicated that both OA and RA patients exhibited pain hypersensitivity (Fig. 1B). In the blood samples, the ratio and count of CD66b^+^ neutrophils among CD45^+^ leukocytes in RA patients were higher than in OA patients and healthy controls (Fig. 1C–E). Additionally, there was no significant difference in the ratio of CD95^+^ or CD206^+^ neutrophils between RA and OA patients (Fig. 1F–I). In the synovial fluid samples, neutrophils accounted for approximately 80 % of total CD45^+^ leukocytes in RA patients, a significantly higher proportion than in OA patients (Fig. 1J–L). Notably, synovial fluid neutrophils in RA patients exhibited a higher ratio of CD95^+^ (N1 state, Fig. 1N–O) and a lower ratio of CD206^+^ (N2 state, Fig. 1Q–R) compared to OA patients. Furthermore, we found that the ratio of neutrophils and their N1 state subset positively correlated with the pain scores of RA patients but not those with OA (Fig. 1M and P). These data suggest that the accumulation and activation of neutrophils in synovial fluid may promote the pathogenesis of RA and RA-associated pain.

### 3.2 GHQ attenuates RA-associated joint swelling and pain

Geranyl hydroquinone (GHQ) is a natural meroterpenoids compound, which is firstly reported by Rudall in 1966 [28] and found in *Aplidium savignyi* [18] and *Ganoderma cochlear* [29]. We employed high-resolution electrospray ionization mass spectrometry (HR-ESI-MS) and nuclear magnetic resonance (NMR) spectroscopy to elucidate the structure of GHQ. As a brown oil, HPLC purity of GHQ was 98 %. The structural characterization revealed the following: HR-ESI-MS *m*/*z* 247.1697 [M + H]^+^ (cacld for C16H23O2: 247.1693); 1H NMR (600 MHz, CDCl3) δ: 6.67 (d, *J* = 8.4 Hz, 1H), 6.61 (d, *J* = 2.9 Hz, 1H), 6.57 (dd, *J* = 8.4, 2.9 Hz, 1H), 5.31 (t, *J* = 6.8 Hz, 1H), 5.08 (t, *J* = 6.8 Hz, 1H), 3.30 (s, 1H), 3.28 (s, 1H), 2.08 (m, 2H), 2.11 (m, 2H), 1.74 (s, 3H), 1.68 (s, 3H), 1.60 (s, 3H); 13C NMR (150 MHz, CDCl3) δ: 149.7, 148.2, 138.4, 132.0, 128.4, 124.0, 121.6, 116.7, 116.6, 113.8, 39.8, 29.7, 26.6, 25.8, 17.8, 16.3. (Figs. S1A–S1D). Emerging studies have indicated the antioxidative properties of GHQ [17,18], prompting this investigation into its potential as a treatment for RA-associated pain. In an *in vitro* model, lipopolysaccharide (LPS) evoked nitric oxide (NO) release in raw264.7 cells, which was reversed by co-incubation of GHQ (Fig. S1E). These results suggest that GHQ may pose anti-inflammatory effects. Therefore, we conducted further investigations into its role in RA-associated pain. Here, the biosafety of GHQ was evaluated using both *in vitro* and *in vivo* methods. HL-60, a human neutrophil cell line, was incubated with GHQ at various doses (0–100 μM) for 6 or 12 h. The cell count and viability were determined using the CCK-8 kit. Our results showed that IC50 of GHQ was 81.29 μM (Fig. S1F). Next, we performed the oral delivery of GHQ at 10 or 50 mg/kg/day on naive mice for 30 consecutive days. The survival ratio, body weight, liver function, and kidney function were not significantly altered by GHQ treatment compared to the vehicle control (Figs. S1G–S1L). Collectively, our results suggest that GHQ is applicable for further investigation at these biosafe doses.

**Fig. 2:**
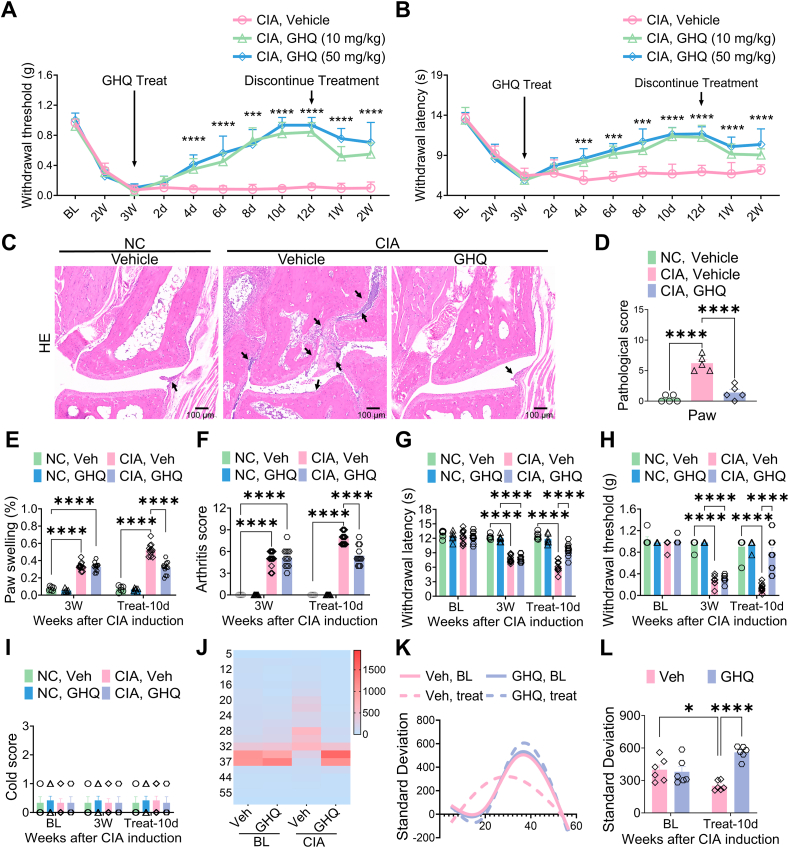
GHQ alleviates RA-associated joint damages and pain. (A–B) Oral administration of GHQ (10 or 50 mg/kg) exerts a persistent analgesic effect in CIA mice. (C–D) HE staining data indicate that GHQ alleviates joint damage in CIA mice, with damages indicated by arrows. Scale bar: 100 μm. (E–F) A 10-day GHQ treatment reduces CIA-induced paw swelling and arthritis scores; however, it does not affect naive mice. (G–I) GHQ treatment alleviated mechanical allodynia and thermal hyperalgesia in CIA mice, while having no effect on naive mice. Additionally, cold allodynia was not observed in CIA mice. (J–L) GHQ treatment reverses the CIA-induced shift in temperature preference in mice, whereas it does not affect naive mice. Data are mean ± SEM. ∗p < 0.05, ∗∗p < 0.01, and ∗∗∗∗p < 0.0001, one-way ANOVA assay followed by Tukey's post hoc test (D), two-way ANOVA assay followed by Tukey's post hoc test (A, B, E, F, G, H, I, L).

We then conducted a 10-day oral administration of GHQ in naive and CIA mice to assess its potential therapeutic effects on RA-associated pain. GHQ treatment (10 or 50 mg/kg/day) alleviated RA-associated mechanical allodynia and thermal hyperalgesia in animals since day 4, with analgesic effects persisting for at least 2 weeks post-treatment (Fig. 2A–B). To evaluate the detailed effects of GHQ, we designated day 10 post-treatment as the experimental endpoint. Hematoxylin and eosin (HE) staining revealed joint damage in the hind paws of CIA mice, which was alleviated by GHQ (Fig. 2C–D). Consistently, GHQ treatment reduced joint swelling in CIA mice compared to the vehicle control (Fig. 2E–F). Further, our behavioral testing demonstrated that GHQ significantly alleviated mechanical allodynia and thermal hyperalgesia in CIA mice compared to the vehicle control. Additionally, cold allodynia was not observed in these CIA mice (Fig. 2G–I). Collagen II immunization significantly altered the thermal preference of animals, which was restored by GHQ treatment (Fig. 2J–L). Collectively, our findings suggest that GHQ may exert beneficial effects on RA-associated inflammation and pain symptoms.

### 3.3 GHQ suppresses neutrophil accumulation under RA condition

**Fig. 3:**
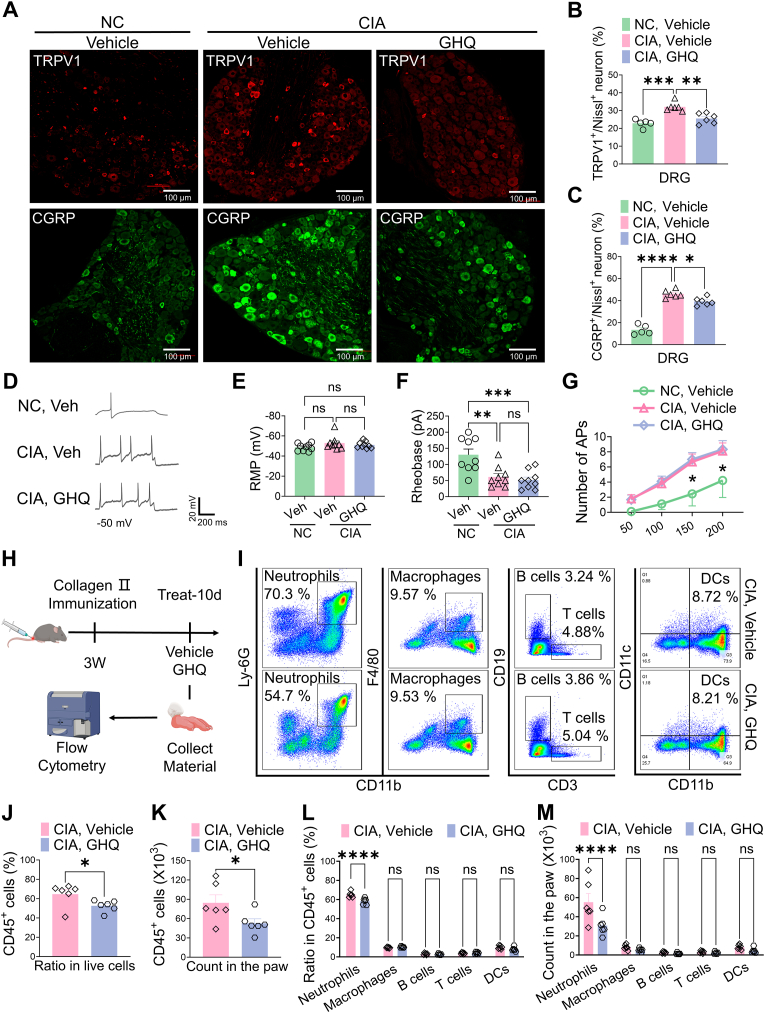
GHQ suppresses neutrophil accumulation under RA condition. (A) Representative IHC images of TRPV1 and CGRP expression in lumbar DRGs. Scale bar: 100 μm. (B–C) IHC data indicate that GHQ reverses CIA-induced TRPV1 and CGRP expression in lumbar DRGs. (D–G) *Ex vivo* recording data indicate that DRG neurons in CIA mice exhibit increased excitability compared to those in naive mice. Additionally, GHQ fails to affect the excitability of TRPV1^+^ neurons in the DRGs of CIA mice. (H) Overview of the experimental protocol. (I) Representative flow cytometry images of leukocytes from the hindpaw samples of CIA mice. (J–K) GHQ decreases the ratio and count of CD45^+^ leukocytes in the hindpaw tissues of CIA mice. (L–M) GHQ reduces the ratio and count of neutrophils, but not of other immune cells, in the hindpaw of CIA mice. Data are mean ± SEM. ∗p < 0.05, ∗∗p < 0.01, and ∗∗∗p < 0.001, one-way ANOVA assay followed by Tukey's post hoc test (B, C, E, F), student *t*-test (J, K), two-way ANOVA assay followed by Bonferroni's post hoc test (G, L, M).

We next investigated the cellular mechanisms underlying the analgesic actions of GHQ against RA-associated pain. First, we assessed whether GHQ could modulate dorsal root ganglion (DRG) sensory neurons. Transient receptor potential vanilloid 1 (TRPV1) is a nonselective ion channel predominantly expressed in pain-related sensory neurons (nociceptors) within the peripheral nervous system. TRPV1 can detect nociceptive stimuli, evoking calcium influx and pain signaling [30]. Calcitonin gene-related peptide (CGRP) is a neuropeptide expressed by peptidergic nociceptors, modulating both immune responses and neuronal activity [31]. Numerous studies indicate that TRPV1 and CGRP serve as markers of peripheral sensitization in DRG neurons associated with RA pain [32,33]. In this study, immunohistochemical data revealed that collagen II immunization significantly increased the expression of TRPV1 and CGRP in DRG neurons of CIA mice treated with vehicle on day 10 compared to controls, suggesting that nociceptors become hypersensitized under RA conditions. Notably, the expression levels of TRPV1 and CGRP were reversed by GHQ treatment, indicating that GHQ may alleviate the hyperexcitability of DRG neurons in these CIA mice (Fig. 3A–C). Next, we generated *Trpv1*^CRE^/*Tdtomato* transgenic mice, in which TRPV1^+^ sensory neurons are labeled with red fluorescence. To determine whether GHQ directly affects neuronal excitability, we established a CIA model in these transgenic mice. After 4 weeks, we prepared DRG tissue slices from the CIA mice and performed electrophysiological recordings to evaluate the effects of GHQ on TRPV1-labeled neurons *ex vivo*. Our observations indicated that DRG neurons in CIA mice exhibited increased excitability compared to those in healthy mice. Furthermore, direct incubation with GHQ (2 min) did not attenuate the hyperexcitability of DRG neurons in CIA mice (Fig. 3D–G). These findings suggest that GHQ may not directly regulate neuronal excitability.

Second, we investigated whether GHQ could regulate immune cells using flow cytometry (Fig. 3H). Our data showed that GHQ treatment downregulated the ratio and count of CD45^+^ leukocytes in the hindpaw samples of CIA mice as compared to the vehicle, suggesting that GHQ may suppress immune cell accumulation in RA joints (Fig. 3I–K). Notably, we found that GHQ significantly decreased the ratio and count of Ly-6G^+^ neutrophils among these CD45^+^ leukocytes, but not affect that of other leukocyte types, such as T cells, B cells DC cells or macrophages (Fig. 3L–M). Together, these findings suggest that GHQ may exert beneficial actions on RA through targeting synovial neutrophils.

### 3.4 GHQ suppresses neutrophil N1 polarization under RA condition

**Fig. 4:**
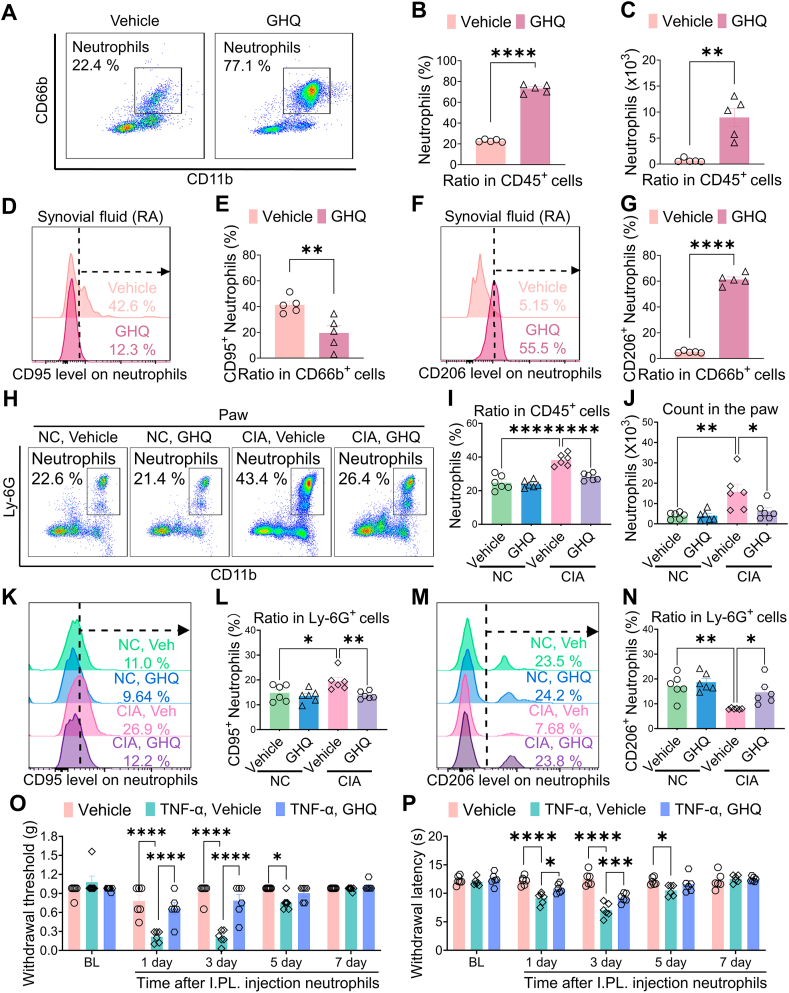
**GHQ suppresses neutrophil N1 polarization under RA condition.** (A) Representative images from flow cytometry analyses of neutrophils in synovial fluid samples from patients with RA. (B, C) GHQ treatment increases the count and ratio of CD66b^+^ neutrophils among CD45^+^ leukocytes. (D–G) GHQ treatment downregulates the ratio of the CD95^+^ subset (D–E) and upregulates that of the CD206^+^ subset (F–G) in synovial fluid neutrophils. (H) Representative images from flow cytometry analyses of neutrophils in the hindpaw samples of CIA mice. (I–J) GHQ reverses collagen immunization-induced neutrophil accumulation in paw tissues in the CIA model. (K–N) GHQ treatment downregulates the ratio of CD95^+^ neutrophils (K–L) and upregulates that of CD206^+^ neutrophils (M − N) in the hindpaw samples of CIA mice. (O–P) Intraplantar adoptive transfer of TNF-α-treated neutrophils evokes mechanical allodynia and thermal hyperalgesia, which is alleviated by co-incubation with GHQ. Data are mean ± SEM. ∗p < 0.05, ∗∗p < 0.01 and ∗∗∗∗p < 0.0001, student *t*-test (B, C, E, G), one-way ANOVA assay followed by Tukey's post hoc test (I, J, L, N) and two-way ANOVA assay followed by Tukey's post hoc test (O, P).

To determine whether GHQ modulates the functional state of joint neutrophils, we examined its effects on the polarization of primary synovial neutrophils harvested from RA patients. Flow cytometry data indicated that GHQ treatment increased the ratio and count of CD66b^+^ neutrophils among the CD45^+^ leukocyte population (Fig. 4A–C). Furthermore, GHQ treatment downregulated the ratio of the CD95^+^ subset and upregulated that of the CD206^+^ subset in these neutrophils *in vitro* (Fig. 4D–G). Given that N1 polarization can accelerate the apoptosis of neutrophils *in vivo* [34], GHQ treatment may prevent the N1 polarization of synovial neutrophils under RA conditions, thereby impeding RA pathogenesis.

To verify this hypothesis, we evaluated the RA-specific therapeutic effects of GHQ using the CIA model. A 10-day oral treatment with GHQ did not affect the ratio and count of neutrophils in the hindpaw samples from naive mice, suggesting that GHQ may not influence immune homeostasis under physiological conditions. Consistent with our previous findings, neutrophil accumulation occurred in the hindpaw samples from CIA mice, which was reversed by GHQ treatment (Fig. 4H–J). Additionally, we found that GHQ did not alter the functional state of neutrophils in hindpaw samples from naive mice. Notably, CIA mice exhibited an increased ratio of CD95^+^ subset and decreased ratio of CD206^+^ neutrophils in hindpaw samples, which was reversed by GHQ treatment (Fig. 4K–N). These data suggest that GHQ may suppress neutrophil accumulation and N1 polarization in CIA mice.

Next, we used the human neutrophil cell line HL-60 to investigate the association between the analgesic and immunoregulatory actions of GHQ. We differentiated HL-60 cells by applying DMSO to acquire the functional characteristics of neutrophils (dHL-60) [35]. DMSO increased the ratio of CD66b^+^ subset among all cultured HL-60 cells (Figs. S2A–S2C). It is well established that the pro-inflammatory cytokine TNF-α is highly expressed in rheumatoid joints, causing immune disorders and promoting RA pathogenesis [36]. Therefore, we incubated these dHL-60 cells with TNF-α to mimic RA conditions *in vitro*. Flow cytometry analysis indicated that TNF-α challenges increased the percentage of CD95^+^ (N1) subset while decreasing the ratio of CD206^+^ (N2) subset, a trend that was reversed by GHQ co-administration (Figs. S2D–S2G). These findings confirm the regulatory role of GHQ on neutrophil polarization under RA conditions. Subsequently, we harvested dHL-60 cells treated with TNF-α or TNF-α/GHQ and performed intraplantar adoptive transfer of these cells into naive recipient mice. Our behavioral testing indicated that TNF-α-treated neutrophils evoked mechanical allodynia and thermal hyperalgesia compared to the vehicle group, suggesting that N1-polarized neutrophils may exhibit pro-nociceptive properties. Importantly, co-incubation with GHQ alleviated pain hypersensitivity induced by the intraplantar adoptive transfer of TNF-α-challenged neutrophils (Fig. 4O–P). Collectively, our data implicate that GHQ alleviates RA-associated pain through suppression of neutrophil N1 polarization.

### 3.5 GHQ target neutrophil ROS pathway under RA condition

**Fig. 5:**
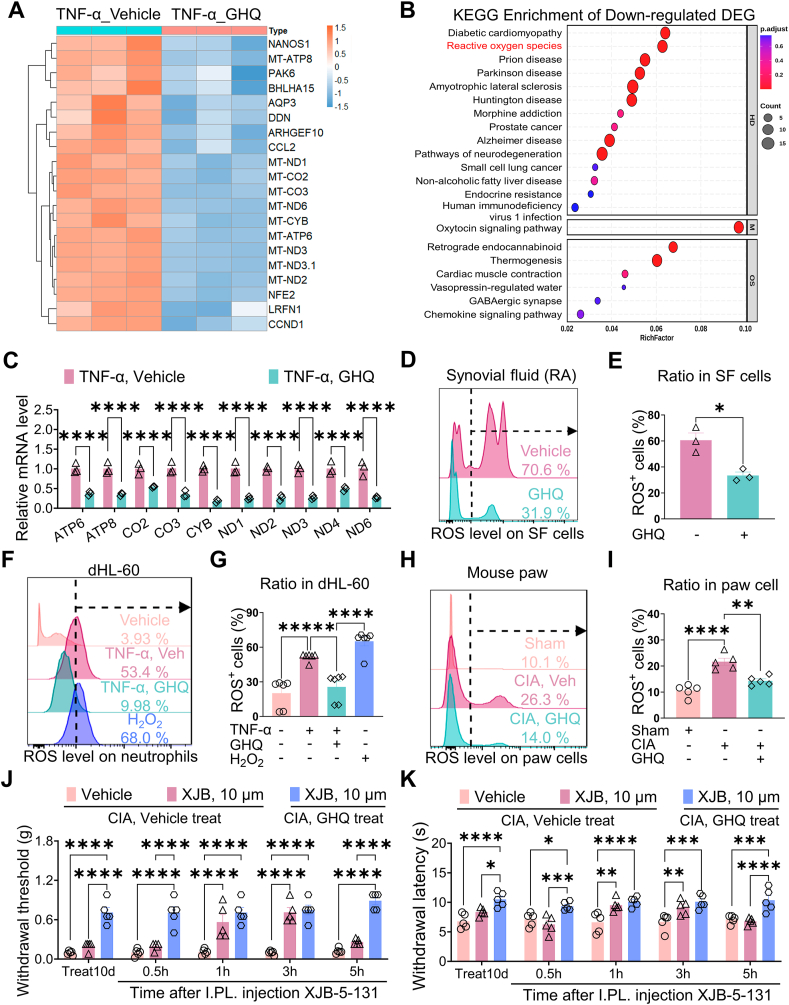
GHQ targets neutrophil ROS pathway under RA condition. (A) RNA-seq analysis indicates that GHQ alters the transcriptome of dHL-60 cells treated with TNF-α. (B) KEGG analysis shows that GHQ suppresses multiple signaling pathways, including the ROS pathway. (C) Real-time PCR data reveal that GHQ downregulates the levels of several genes associated with ROS production. (D–E) GHQ incubation decreases the ratio of ROS ^+^ cells in the synovial fluid of RA patients *in vitro*. (F–G) GHQ incubation further decreases the ratio of ROS^+^ cells in TNF-α-challenged dHL-60 cells *in vitro*. (H–I) GHQ treatment reduces the ratio of ROS ^+^ cells in the hindpaw tissues of CIA mice *in vivo*. (J–K) The ROS scavenger XJB-5-131 increases mechanical and thermal pain thresholds in CIA mice treated with the vehicle, but not in those treated with GHQ. Data are mean ± SEM. ∗p < 0.05, ∗∗p < 0.01, ∗∗∗p < 0.001 and ∗∗∗∗p < 0.0001, two-way ANOVA assay followed by Tukey's post hoc test (C, J, K), student *t*-test (E), one-way ANOVA assay followed by Tukey's post hoc test (G, I).

In this study, we investigated the molecular mechanisms underlying the analgesic action of GHQ. We collected mRNA from dHL-60 cells from the experimental groups described in Fig. S2 and performed RNA-seq to analyze their transcriptomic profiles. The dHL-60 cells were validated for the expression profiles of human neutrophil-specific genes [37,38] (Figs. S3A–S3B). We found that GHQ upregulated the mRNA levels of 558 genes and downregulated those of 270 genes (Fig. 5A, Fig. S3C). KEGG enrichment analysis and GO analysis indicated that multiple pathways, including the reactive oxygen species (ROS) pathway, were downregulated by GHQ (Fig. 5B, Fig. S3D). Real-time PCR data confirmed that the transcriptional levels of several genes associated with ROS production, including *ATP6*, *ATP8*, *CO2* and so on, were decreased following GHQ treatment (Fig. 5C). These findings suggest that GHQ may target neutrophil ROS pathway to exert its biological functions.

To verify this hypothesis, we performed flow cytometry to evaluate ROS production in neutrophils under our experimental conditions. Our data indicated that *in vitro* incubation with GHQ (6 h) decreased the ratio of ROS^+^ subset of synovial neutrophils harvested from RA patients (Fig. 5D–E). Consistently, TNF-α challenge increased the ratio of ROS^+^ dHL-60 cells, which was reversed by co-incubation with GHQ. As a positive control, H_2_O_2_ incubation promoted ROS production in these dHL-60 cells (Fig. 5F–G). Furthermore, we demonstrated that collagen II immunization increased ROS production *in vivo*, as evidenced by the elevated ratio of ROS^+^ cells in the hindpaw samples. Notably, GHQ treatment significantly reversed CIA-evoked ROS production in mice (Fig. 5H–I). On day 10 after vehicle treatment, intraplantar injection of ROS-targeted scavenger XJB-5-131 alleviated mechanical allodynia and thermal hyperalgesia in CIA mice, suggesting that the ROS pathway contributes to RA-associated pain. Furthermore, XJB-5-131 did not increase the pain thresholds of CIA mice receiving GHQ treatment (Fig. 5J–K). Taken together, our *in vitro* and *in vivo* evidence suggest that the RA-specific analgesic action of GHQ depends on the ROS pathway in neutrophils.

### 3.6 GHQ targets MGST3 to suppress ROS production in neutrophils

**Fig. 6:**
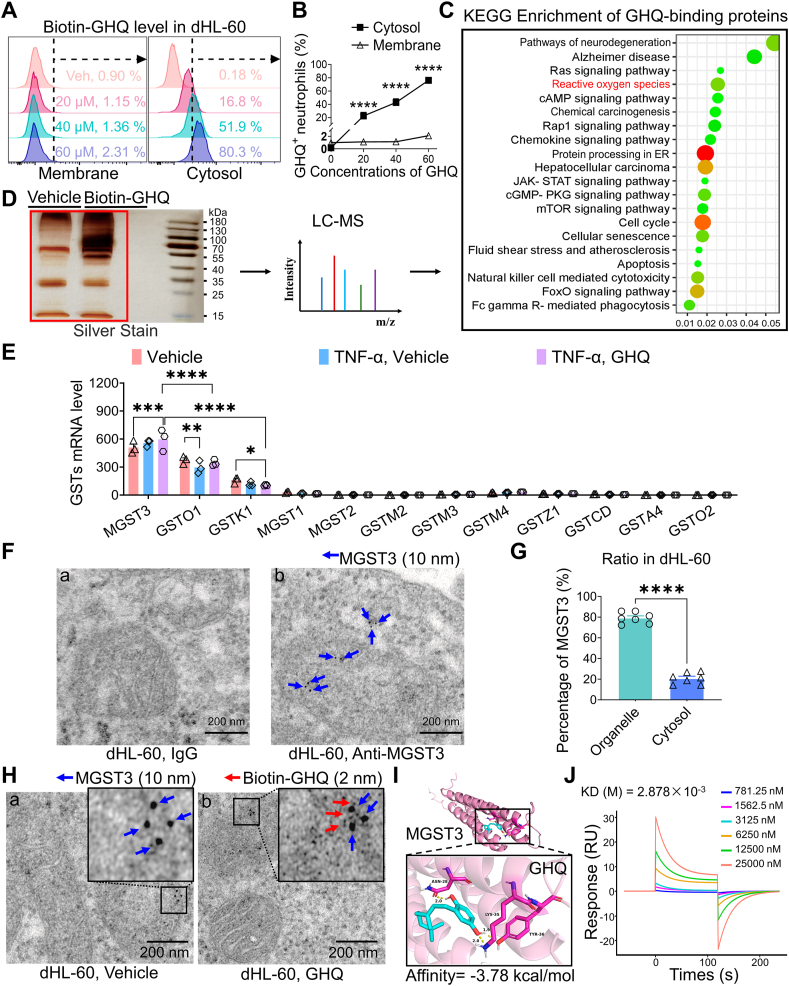
GHQ binds to MGST3 in neutrophils. (A–B) Flow cytometry analysis is conducted to assess the localization of the GHQ probe in the membrane and cytoplasm of dHL-60 cells. (C–D) LC-MS/MS identifies more than 200 protein candidates targeted by GHQ. (E) RNA-seq data show the expression of GSTs in dHL-60 cells treated with either vehicle, TNF-α, or a combination of TNF-α and GHQ. (F–G) IEM images indicate that MGST3 is predominantly localized in the organelles of dHL-60 cells. Scale bar: 200 nm. (H) The IEM data reveal the co-localization of MGST3 and GHQ in dHL-60 cells. Scale bar: 200 nm. (I) Molecular docking analysis predicts that GHQ binds to MGST3. (J) SPR analysis of the binding affinity of GHQ to recombinant human MGST3. Data are mean ± SEM. ∗p < 0.05, ∗∗p < 0.01, ∗∗∗p < 0.001 and ∗∗∗∗p < 0.0001, two-way ANOVA assay followed by Tukey's post hoc test (B, E), student *t*-test (G).

To identify the potential binding targets of GHQ in neutrophils, we designed and synthesized a GHQ probe with a biotin tag. We incubated dHL-60 cells containing the GHQ probe with FITC-avidin antibody under both permeabilized and non-permeabilized conditions. Interestingly, flow cytometry data indicated that the intracellular level of the GHQ probe increased in a dose-dependent manner, whereas the GHQ probe on the plasma membrane was rarely detected (Fig. 6A–B). Subsequently, GHQ-labeled proteins were purified from the lysates of dHL-60 cells using streptavidin beads and enriched by electrophoresis on SDS-PAGE gel (Fig. 6D). LC-MS/MS identified more than 200 candidate proteins, as shown in the KEGG enrichment analysis (Fig. 6C). Among these candidates, microsomal glutathione S-transferase 3 (MGST3), a member of the glutathione S-transferase (GST) family, is well known for its inhibitory role in ROS production [39]. RNA-seq analysis revealed that MGST3 was highly expressed in neutrophils compared to other members of the GST family. Notably, co-incubation with GHQ/TNF-α resulted in a significant upregulation of MGST3 expression compared to dHL-60 cells treated with a vehicle (Fig. 6E). Immunoelectron microscopy data indicated that MGST3 (indicated by 10 nm colloidal gold particles) was predominantly located in membrane-bound organelles (Fig. 6F–G). Furthermore, some GHQ molecules (indicated by 2 nm colloidal gold particles) were found close to MGST3 (Fig. 6H). As the 3D structure of human MGST3 remains unavailable, we predicted it using AlphaFold2. Our molecular docking analysis suggested that GHQ binds to the MGST3 monomer (Fig. 6I). Additionally, surface plasmon resonance (SPR) analysis demonstrated that GHQ exhibited binding affinity for recombinant human MGST3 (Fig. 6J).

**Fig. 7:**
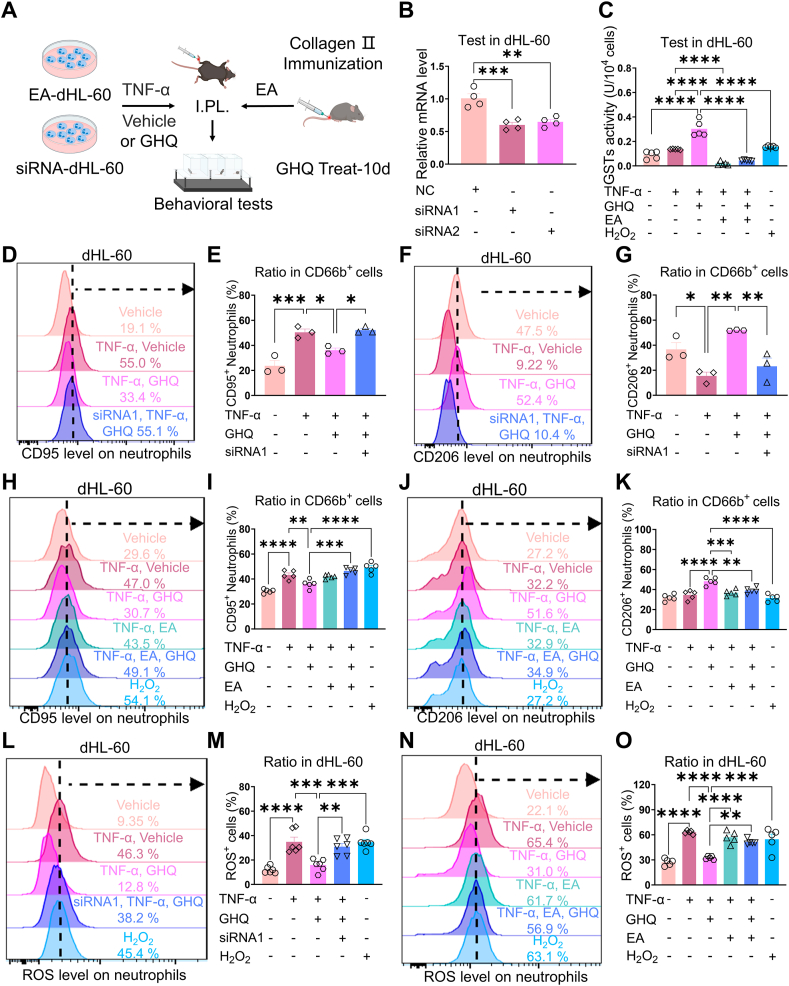
RA-specific anti-inflammatory and antioxidative actions of GHQ require MGST3 in neutrophils. (A) Diagram of the experimental protocol. (B) Real-time PCR analysis indicates that two sets of *MGST3* siRNA effectively knock down *MGST3* expression in dHL-60 cells. (C) GHQ enhances the enzymatic activity of GST in dHL-60 cells under TNF-α challenge. (D–K) Flow cytometry data show that co-administration of GHQ reverses the TNF-α-induced increase in the ratio of the CD95^+^ subset and the decrease in the ratio of the CD206 subset in dHL-60 cells. Notably, pre-application of either (D–G) *MGST3* siRNA1 or (H–K) etacrynic acid (EA) abolishes the anti-inflammatory effects of GHQ. (L–O) TNF-α challenge evokes ROS production in dHL-60 cells, which is reversed by co-incubation with GHQ. Importantly, co-application of *MGST3* siRNA1 or etacrynic acid (EA) abolishes the antioxidative action of GHQ *in vitro*. Data are mean ± SEM. ∗p < 0.05, ∗∗p < 0.01, ∗∗∗p < 0.001 and ∗∗∗∗p < 0.0001, one-way ANOVA assay followed by Tukey's post hoc test (B, C, E, G, I, K, M and O).

Next, we investigated whether MGST3 is involved in GHQ-mediated regulation of ROS production (Fig. 7A). Two sets of *MGST3*-targeting siRNA sequences were designed. Real-time PCR data confirmed the knockdown of MGST3 expression in naive dHL-60 cells by siRNA1 and siRNA2, respectively (Fig. 7B). As MGST3-targeted chemical agents are not commercially available, we utilized the pan-GST inhibitor etacrynic acid (EA) [40]. In the GST assay, we found that GHQ enhanced GST enzyme activity in dHL-60 cells under TNF-α challenge, an effect that was abolished by EA incubation, suggesting a modulatory role of GHQ on MGST3 function (Fig. 7C). As described previously, co-incubation of GHQ reversed the TNF-α-evoked increase in the ratio of the CD95^+^ subset and the decrease in the ratio of the CD206^+^ subset in dHL-60 cells. Notably, either *MGST3* siRNA1 (Fig. 7D–G) or EA (Fig. 7H–K) abolished the GHQ-mediated effect on neutrophil polarization. Furthermore, co-incubation with siRNA1 or EA abolished the GHQ-mediated inhibition of ROS production in dHL-60 cells (Fig. 7L–O). Collectively, these data suggest that GHQ requires MGST3 to suppress N1 polarization and ROS production in neutrophils.

**Fig. 8:**
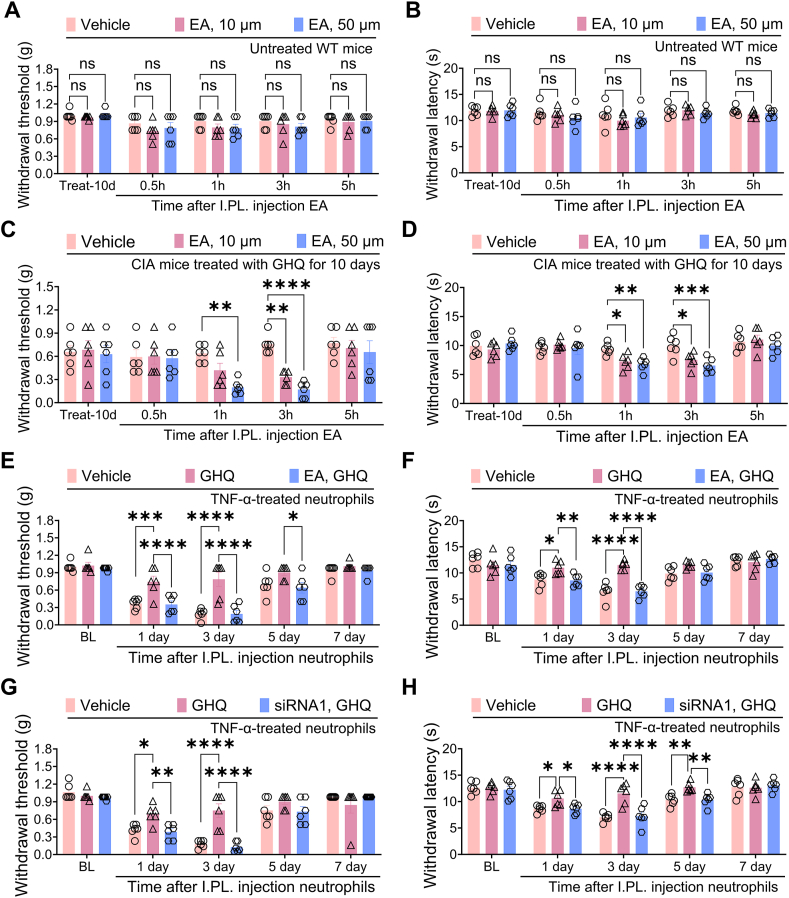
RA-specific analgesic action of GHQ requires MGST3 in neutrophils. (A–B) A single dose intraplantar injection of EA fails to induce any mechanical (A) or thermal (B) pain in naive mice. (C–D) Intraplantar injection of etacrynic acid (EA) evokes acute mechanical allodynia and thermal hyperalgesia in GHQ-treated CIA mice in a dose-dependent manner. (E–H) Adoptive transfer of TNF-α-challenged dHL-60 cells induces mechanical allodynia and thermal hyperalgesia in naive recipient mice, which is reversed by co-incubation with GHQ. Furthermore, co-application of EA (E–F) or *MGST3* siRNA1 (G–H) abolishes the analgesic effects of GHQ in recipient mice. Data are mean ± SEM. ∗p < 0.05, ∗∗p < 0.01, ∗∗∗p < 0.001 and ∗∗∗∗p < 0.0001, two-way ANOVA assay followed by Tukey's post hoc test (A–H).

We also examined the role of the GHQ/MGST3 pathway in neutrophil-mediated pain processing. In naive mice, EA did not affect mechanical or thermal pain thresholds, suggesting that the GST pathway may not modulate physiological pain (Fig. 8A–B). As described earlier, CIA mice developed mechanical allodynia and thermal hyperalgesia, which were alleviated by a 10-day oral treatment with GHQ. Notably, an acute intraplantar injection of EA transiently decreased the paw withdrawal threshold and latency in GHQ-treated CIA mice in a dose-dependent manner, indicating that blocking GSTs may abolish the RA-specific analgesic effect of GHQ (Fig. 8C–D). Furthermore, intraplantar adoptive transfer of TNF-α-challenged dHL-60 cells evoked persistent mechanical allodynia and thermal hyperalgesia in naive recipient mice, effects that were attenuated by co-administration of GHQ. Importantly, co-incubation with EA (Fig. 8E–F) or *MGST3* siRNA (Fig. 8G–H) abolished the GHQ-mediated analgesic effects in these recipient mice. Together, our study implicates that GHQ requires MGST3 to alleviate RA-associated pain.

### 3.7 GHQ exhibits superior therapeutic actions on collagen-induced rheumatoid arthritis than methotrexate

Methotrexate has been a first-line DMARD since the 1960s and is currently used by an estimated 900,000 patients in the United States [6]. However, methotrexate is associated with several detrimental side effects, including gastrointestinal symptoms, lung complications, and susceptibility to infections [6]. In this study, we compared the therapeutic effects of GHQ and methotrexate using the CIA model. In our experimental design, methotrexate was orally administered to CIA mice following the same protocol as GHQ treatment. HE staining data indicated that both GHQ and methotrexate alleviated joint damage in CIA mice. Notably, mice receiving GHQ treatment exhibited a lower pathological score than those treated with methotrexate (Figs. S4A–S4B). Consistent with this, methotrexate demonstrated therapeutic effects on CIA mice, as evidenced by reduced paw swelling, lower arthritis index scores, and increased pain thresholds. Of note, GHQ treatment showed superior therapeutic effects in managing paw swelling and pain hypersensitivity in CIA mice compared to methotrexate (Figs. S4C–S4F). Furthermore, collagen II immunization evoked ROS production in the hindpaw samples of CIA mice, which was significantly reversed by GHQ but not by methotrexate (Figs. S4G–S4H). Additionally, the ratio of ROS^+^ cells in the hindpaw samples of CIA mice treated with vehicle or methotrexate, but not GHQ, negatively correlated with pain threshold and latency, suggesting that the RA-specific therapeutic effect of methotrexate does not rely on antioxidative action (Figs. S4I–S4N). Collectively, these findings suggest that GHQ may provide greater efficacy and fewer cytotoxic effects in treating RA compared to methotrexate.

## 4 Discussion

Chronic pain is a significant characteristic of RA, posing a substantial health burden on patients worldwide [2]. However, the underlying mechanisms remain poorly understood. Previously, we have demonstrated that crosstalk between immune cells and peripheral sensory neurons contributes to the pathogenesis of pathological pain [[11], [12], [13], [14],^27^,[41], [42], [43]]. Neutrophils are the first responders and the most abundant cell type within the RA joint [7,9,10]. Emerging evidence indicates the polarization of neutrophils promotes the pathology of various diseases [8]. Currently, it remains unclear whether and how neutrophils contribute to RA-associated pain. Thus, this study identifies a neutrophil mechanism underlying RA-associated joint inflammation and pain. Further, our study shows that geranyl hydroquinone (GHQ) alleviates RA-associated pain through suppressing neutrophil-induced inflammation.

Our research indicates that synovial neutrophils modulate RA-associated pain through the MGST3/ROS pathway. In affected synovium, the accumulation of reactive oxygen species (ROS) causes mitochondrial damage, leading to inflammatory responses. Thus, ROS is considered an indirect indicator of disease severity [44]. The production and release of ROS is a critical function and hallmarker of pro-inflammatory N1 neutrophils under pathological conditions [9,10]. Since neutrophils are the most numerous leukocytes in synovial fluid, they are regarded as a major source of ROS in the synovium of RA patients [9]. In this study, our data suggest that neutrophil-derived ROS is involved in the pathology of RA-associated pain. Glutathione serves as an essential redox buffer in cells and is required for ROS scavenging [45]. Glutathione S-transferases (GSTs), present in almost all cell types, catalyze the conjugation of glutathione, thereby exerting antioxidative actions [39]. Impairment of components in the GST family may lead to increased ROS production in neutrophils under various disease conditions, including RA [46]. By analyzing data from the recently published transcriptomic database (GSE116899), we find that *MGST3* expression in synovial neutrophils is downregulated under RA conditions. In this study, our evidence suggests that MGST3 is an essential regulator of neutrophil ROS production and a potential target for RA treatment.

This study further demonstrates that GHQ treatment exerts RA-specific therapeutic actions and uncovers several underlying cellular and molecular mechanisms. First, GHQ interferes with the accumulation and N1 polarization of neutrophils in synovial fluid from RA patients and paw tissues from CIA mice. Second, GHQ suppresses RA-evoked ROS production in synovial neutrophils from RA patients, TNF-α-challenged dHL-60 cells, and hindpaw tissues from CIA mice. Third, GHQ requires MGST3 to suppress ROS production in TNF-α-challenged dHL-60 cells. Fourth, GHQ requires MGST3 to alleviate RA associated pain hypersensitivity in mice.

Despite the promising results, there are several limitations to this research. (1) Although our electrophysiological data indicate that acute incubation with GHQ fails to directly modulate the excitability of TRPV1^+^ DRG neurons, it will be of great interest to investigate whether GHQ may exert long-term effects on sensory neurons by altering their transcriptome (e.g., by inhibiting CGRP expression). (2) In this study, we found that GHQ requires MGST3 to regulate ROS production in neutrophils. However, the expression and functional patterns of MGST3 in neutrophils in the context of RA pathology remain unclear and warrant further research. (3) Our LC-MS/MS data suggest that GHQ exhibits strong binding affinity for MGST3 in neutrophils. Further analysis indicates that the antioxidative action of GHQ requires MGST3. However, molecular docking and SPR analysis imply that GHQ may show weak binding affinity for the MGST3 monomer. Mammalian cytosolic GSTs form homogeneous or heterogeneous dimers to exert their biological functions [47], suggesting that the MGST3 dimer or complex may exhibit increased affinity for GHQ compared to its monomer. Thus, mechanism underlying the interaction between MGST3 and GHQ requires additional investigation. (4) As this study examines the therapeutic effects and underlying mechanisms of GHQ treatment using male animals, further investigation is needed with female subjects.

In summary, our results demonstrate that the accumulation and N1 polarization of synovial neutrophils drive RA-associated pain, representing a critical advancement in our understanding of RA pathology. Importantly, GHQ exerts RA-specific analgesic effects by inhibiting neutrophil N1 polarization through the MGST3/ROS pathway. These findings provide mechanistic insights into the pathology of RA-associated pain and support the development of new pain management strategies for RA patients.

## CRediT authorship contribution statement

**Sen Huang:** Writing – review & editing, Validation, Investigation, Data curation. **Yuxin Xie:** Validation, Investigation. **Zhaochun Zhan:** Validation, Investigation. **Fengdong Liu:** Investigation. **Peiyang Liu:** Investigation. **Fei Xu:** Investigation. **Tingting Xu:** Investigation. **Zhenning Fang:** Investigation. **Zhiqiang Chen:** Investigation. **Qingjian Han:** Supervision, Resources, Funding acquisition. **Ligang Jie:** Resources. **Rougang Xie:** Supervision, Funding acquisition. **Hongfei Zhang:** Supervision, Resources. **Shiyuan Xu:** Supervision, Resources. **Yiwen Zhang:** Writing – review & editing, Supervision, Funding acquisition. **Kai Mo:** Writing – review & editing, Supervision, Funding acquisition. **Xin Luo:** Writing – review & editing, Writing – original draft, Supervision, Resources, Funding acquisition, Conceptualization.

## Resource availability

Requests for further information should be directed to and will be fulfilled by the lead contact, Xin Luo (xin.luo21@hotmail.com). This project did not generate new unique reagents or new materials. Data reported in this paper will be shared by the lead contact upon request. This paper does not report the original code.

## Declaration of competing interest

The authors have declared no conflict of interest.

## Data Availability

Data will be made available on request.
