# Supplementary material for: Geranyl hydroquinone alleviates rheumatoid arthritis-associated pain by suppressing neutrophil accumulation, N1 polarization and ROS production in mice
Source: Redox Biol. 2025 Mar 18;82:103603. doi: 10.1016/j.redox.2025.103603 (PMC11986610; doi:10.1016/j.redox.2025.103603)
Supplement: Multimedia component 1 [file mmc1.docx]

**Supplementary Material**

**Supplementary figures (Figs. S1-S4)**

**
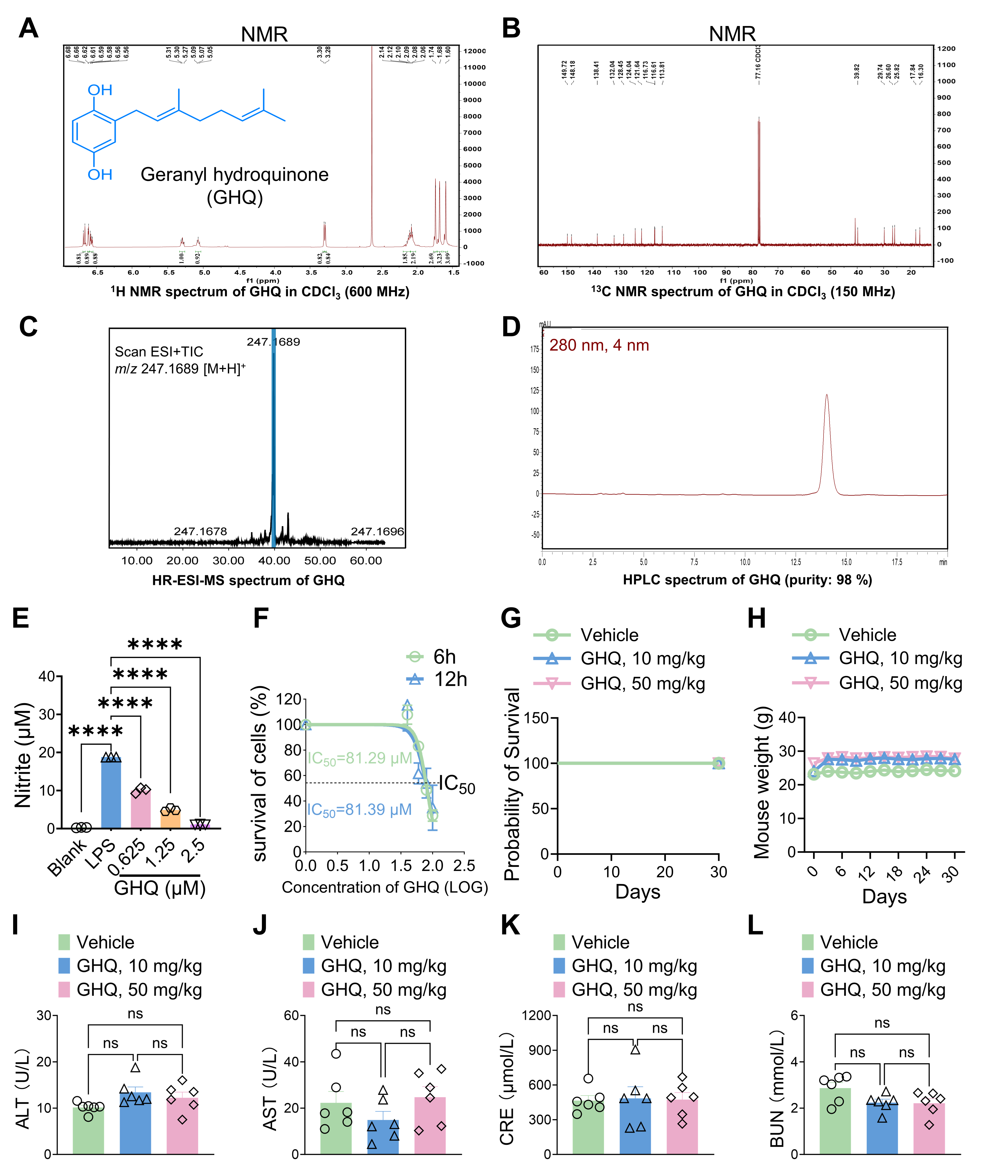
**

**Figure S1.** **Structure, anti-inflammatory effect and biosafety of GHQ.**

(A-D) NMR, HR-ESI-MS and HPLC analyses are employed to determine the structure of GHQ. (E) GHQ reverses LPS-evoked NO release in raw 264.7 cells *in vitro*. (F) The CCK-8 assay indicates that the IC50 of GHQ is 81.29 μM in HL-60 cells. (G-L) Oral administration of GHQ at doses of 10 or 50 mg/kg to naive mice for 30 consecutive days does not result in any significant differences in (G) survival ratio, (H) body weight, or (I-J) liver or (K-L) kidney function compared to the vehicle group. Data are mean ± SEM. *p < 0.05, **p < 0.01, and ***p < 0.001, one-way ANOVA assay followed by Tukey's post hoc test (E, I, J, K, L) and two-way ANOVA assay followed by Tukey's post hoc test (G, H).


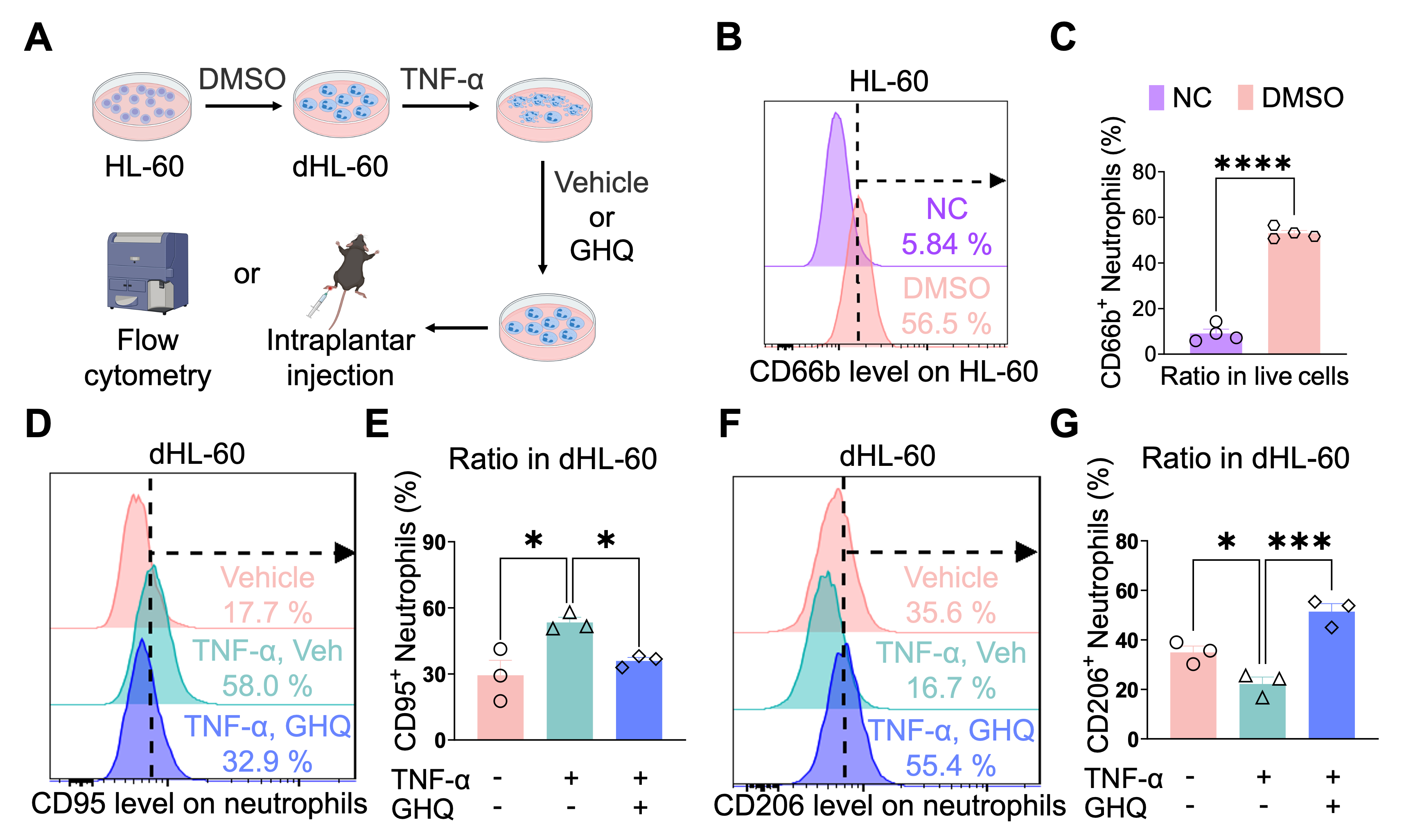


**Figure S2. GHQ reverses TNF-**α**-evoked neutrophil polarization.**

(A) Diagram of the experimental protocol. (B-C) DMSO increases the ratio of the CD66b^+^ subset among all cultured HL-60 cells. (D-E) Flow cytometry data indicate that TNF-α challenge increases the percentage of the CD95^+^ subset of dHL-60 cells, which is reversed by co-administration of GHQ. (F-G) Co-incubation with GHQ increases the ratio of the CD206^+^ (N2) subset compared to the TNF-α treatment group. Data are mean ± SEM. *p < 0.05, **p < 0.01, ***p < 0.001 and ****p < 0.0001, student t test (C), one-way ANOVA assay followed by Tukey's post hoc test (E, G).

**
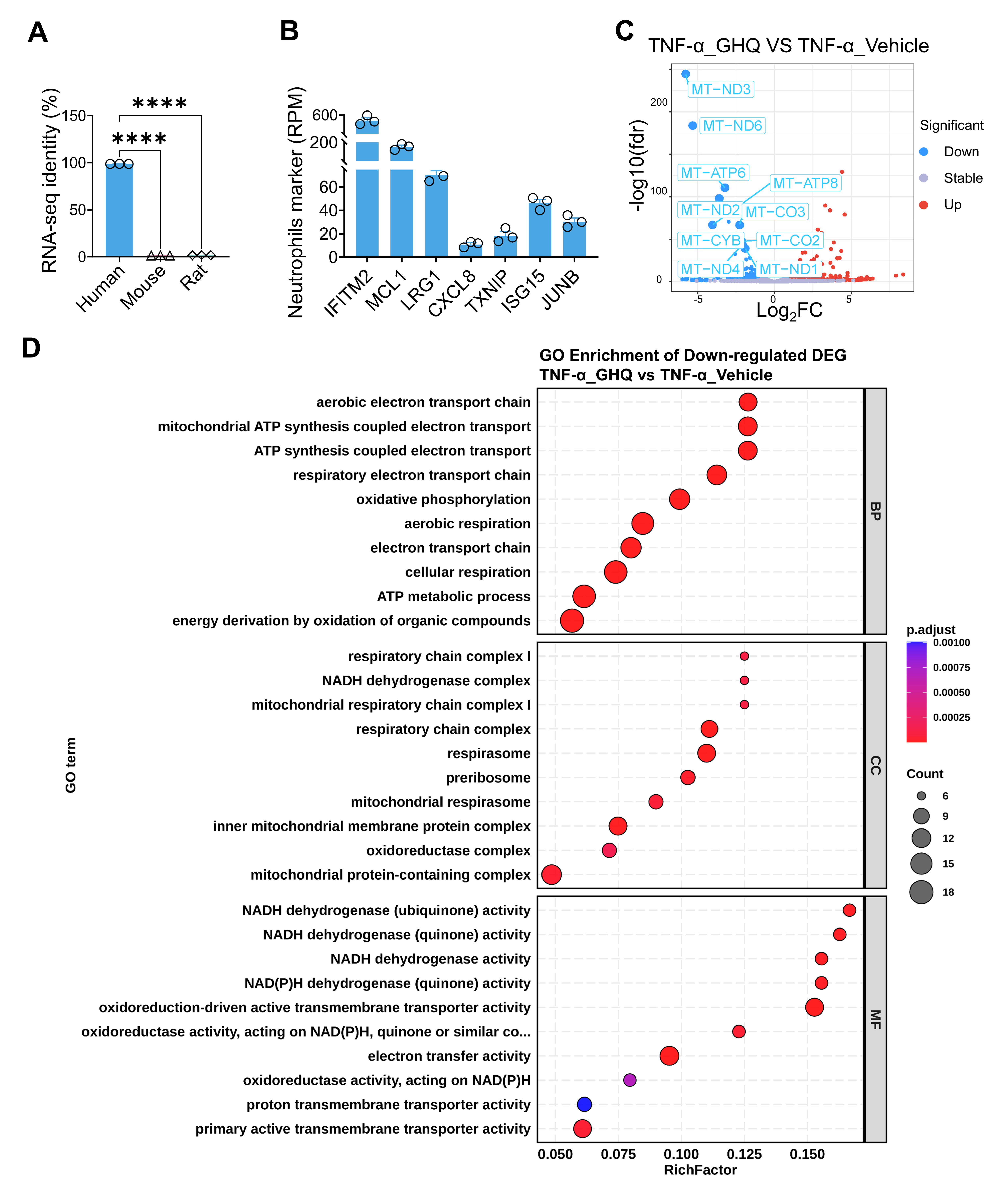
**

**Figure S3. GHQ targets the ROS pathway in neutrophils.**

(A-B) RNA-seq data indicate that dHL-60 cells express genes specific to human neutrophils. (C) GHQ incubation upregulates the mRNA levels of 558 genes and downregulates those of 270 genes in TNF-α-challenged dHL-60 cells. (D) GO analysis indicates that GHQ attenuates the ROS signaling pathway. Data are mean ± SEM. ****p < 0.0001, one-way ANOVA assay followed by Tukey's post hoc test (A).

**
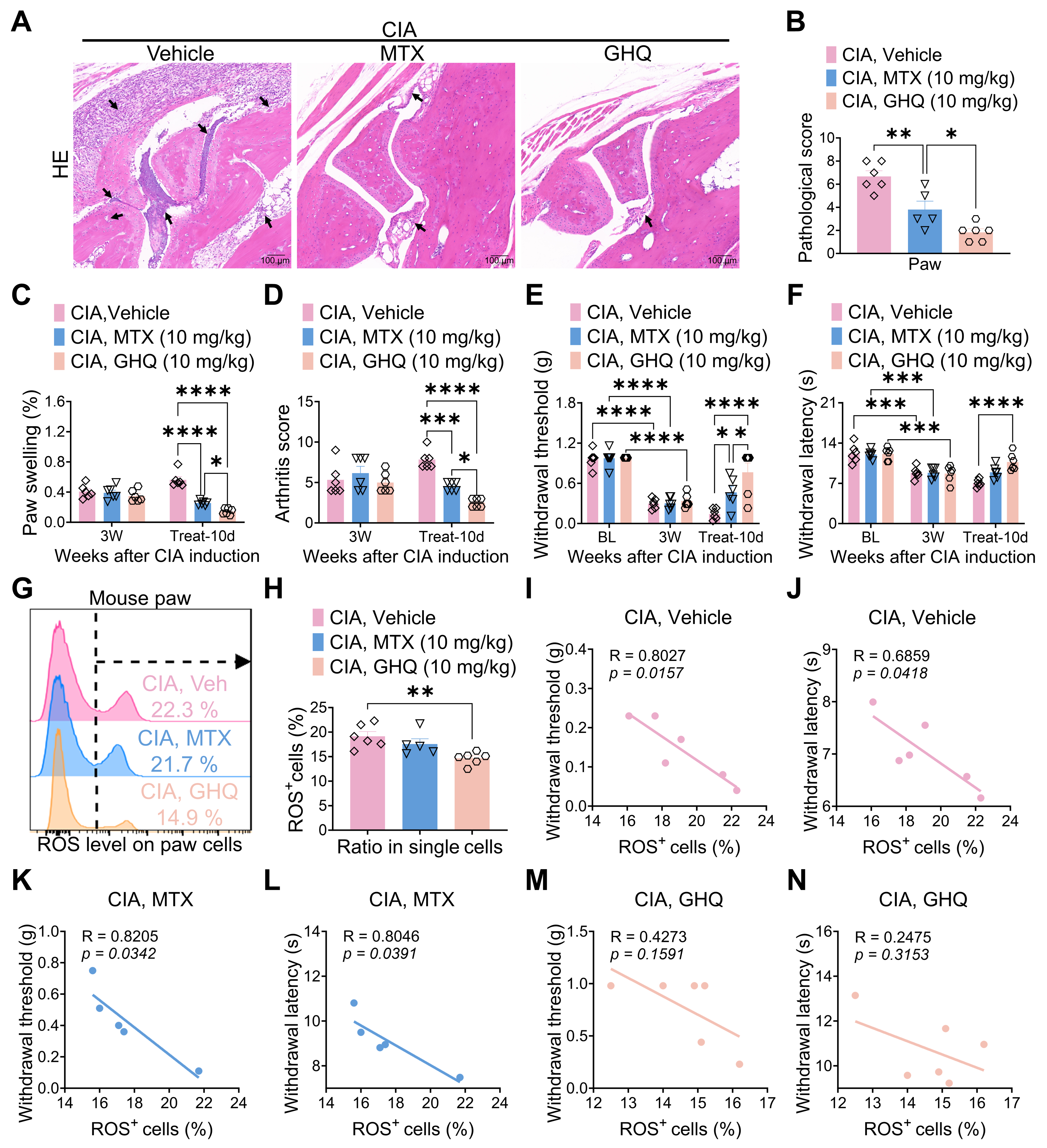
**

**Figure S4.** **GHQ exhibits superior therapeutic effects than methotrexate (MTX) under RA condition.**

(A-B) HE staining data indicate that both GHQ and MTX alleviate joint damage in CIA mice, damage sites indicated by arrows. Scale bar: 100 μm. (B-D) GHQ demonstrates greater therapeutic efficacy in reducing (B) joint damages, (C) paw swelling and (D) arthritis scores in CIA mice compared to methotrexate. (E-F) GHQ shows enhanced analgesic effects in CIA mice relative to methotrexate. (G-H) GHQ, but not methotrexate, decreases the ratio of ROS^+^ cells in the hindpaw tissues of CIA mice. (I-N) In the vehicle and methotrexate groups, the ratio of ROS^+^ cells negatively correlates with mechanical and thermal pain thresholds. Such correlation is not observed in the GHQ group. Data are mean ± SEM. *p < 0.05, **p < 0.01, ***p < 0.001 and ****p < 0.0001, one-way ANOVA assay followed by Tukey's post hoc test (B, H), two-way ANOVA assay followed by Tukey's post hoc test (C, D, E, F), Pearson correlation analysis (I, J, K, L, M, N).
