# Supplementary material for: Geranyl hydroquinone alleviates rheumatoid arthritis-associated pain by suppressing neutrophil accumulation, N1 polarization and ROS production in mice
Source: Redox Biol. 2025 Mar 18;82:103603. doi: 10.1016/j.redox.2025.103603 (PMC11986610; doi:10.1016/j.redox.2025.103603)
Supplement: Multimedia component 2 [file mmc2.docx]

**Supplementary Material**

**Supplementary tables (Tables. S1-S4)**

**Table S1. Characteristics of all participants in our study**

| ID | Diagnosis | VAS |
| --- | --- | --- |
| HC1 | Healthy | 0 |
| HC2 | Healthy | 0 |
| HC3 | Healthy | 0 |
| HC4 | Healthy | 0 |
| HC5 | Healthy | 0 |
| HC6 | Healthy | 0 |
| OA1 | Osteoarthritis | 35 |
| OA2 | Osteoarthritis | 22 |
| OA3 | Osteoarthritis | 65 |
| OA4 | Osteoarthritis | 21 |
| OA5 | Osteoarthritis | 35 |
| OA6 | Osteoarthritis | 53 |
| OA7 | Osteoarthritis | 18 |
| OA8 | Osteoarthritis | 26 |
| RA1 | Rheumatoid arthritis | 38 |
| RA2 | Rheumatoid arthritis | 33 |
| RA3 | Rheumatoid arthritis | 68 |
| RA4 | Rheumatoid arthritis | 75 |
| RA5 | Rheumatoid arthritis | 56 |
| RA6 | Rheumatoid arthritis | 48 |
| RA7 | Rheumatoid arthritis | 82 |
| RA8 | Rheumatoid arthritis | 72 |

**Table S2. A list of reagents for this study**

| Reagent or resource | Source | Identifier |
| --- | --- | --- |
| Chemicals, peptides, and recombinant proteins | | |
| Geranyl hydroquinone | RR SCIENTIFIC | R100832 |
| Human TNF-alpha Recombinant Protein | Thermofisher | 300-01A-1MG |
| Invitrogen Lipofectamine 2000 | Thermofisher | 11668030 |
| Methotrexate | GLPBIO | GC10405 |
| Ethacrynic acid | MCE | HY-B1640 |
| H2DCFDA | MCE | HY-D0940 |
| Streptavidin Magnetic Beads | MCE | HY-K0208 |
| Protease Inhibitor Cocktail | MCE | HY-K0010 |
| Phosphatase Inhibitor Cocktail Ⅱ | MCE | HY-K0022 |
| Penicillin-Streptomycin Liquid | Solarbio | P1400 |
| RPMI Medium 1640 | Solarbio | 10491 |
| DMEM | Solarbio | 11995 |
| Red Blood Cell Lysis Buffer | Solarbio | R1010 |
| SDS-PAGE Gel Kit | Solarbio | P1200 |
| Glutathione S-transferase(GST) Activity Assay Kit | Solarbio | BC0355 |
| Cell lysis buffer for Western and IP | beyotime | P0013 |
| Easy-Load™ Multiplex PCR Master Mix | beyotime | D7305M |
| FreeZol Reagent | Vazyme | R711-01 |
| Urea Assay Kit | Nanjing jiancheng Bioengineering Institute | C013-2-1 |
| Creatinine (Cr) Assay kit ( sarcosine oxidase ) | Nanjing jiancheng Bioengineering Institute | C011-2-1 |
| Alanine aminotransferase Assay Kit | Nanjing jiancheng Bioengineering Institute | C009-2-1 |
| Aspartate aminotransferase Assay Kit | Nanjing jiancheng Bioengineering Institute | C010-3-1 |
| Software and algorithms | | |
| GraphPad Prism v9.5.1 | GraphPad Software | <https://www.graphpad.com/> |
| Flowjo v10.8.1 | Flowjo v10 | <https://www.flowjo.com/solutions/flowjo> |
| R (version 4.4.1) | R Development Core Team | <https://cran.r-project.org/> |
| ImageJ 1.53t | Wayne Rasband and contributors National Institutes of Health, USA | <http://imagej.nih.gov/ij> |
| Gradient 1.51.1.16 | BIOSEB | [http://www.bioseb.com](http://www.bioseb.com/) |
| PEAKS Studio 8.5 | Bioinformatics Solutions Inc | <https://www.bioinfor.com/peaks-85-release/> |
| NIS-Elements Viewer 5.21.00 | Nikon | <https://www.microscope.healthcare.nikon.com/> |
| Discovery Studio 2021 | BIOVIA | <https://www.3ds.com/> |
| Pyrx-0.8 | SOURCEFORGE | <https://pyrx.sourceforge.io/> |
| AutoDock Vina39 | Molecular Biology Building | <https://vina.scripps.edu/> |

**Table S3. The sequence of primers for Real-time PCR**

| Genes |  | Sequence (5'→3') |
| --- | --- | --- |
| For Real-time PCR |  |  |
| human MT-ND1 | Forward | CGGGCTACTACAACCCTTCG |
|  | Reverse | AGGAGGCCTAGGTTGAGGTT |
| human MT-ND2 | Forward | GCAAGCAACCGCATCCATAA |
|  | Reverse | GGTGCCTTGGGTAACCTCTG |
| human MT-ND3 | Forward | GCGGCTTCGACCCTATATCC |
|  | Reverse | AGGGCTCATGGTAGGGGTAA |
| human MT-ND4 | Forward | ACAAGCTCCATCTGCCTACG |
|  | Reverse | GAAGCTTCAGGGGGTTTGGA |
| human MT-ND6 | Forward | AATCATACAAAGCCCCCGCA |
|  | Reverse | TGGGGTTAGCGATGGAGGTA |
| human MT-ATP6 | Forward | AGGCACACCTACACCCCTTA |
|  | Reverse | TATTGCTAGGGTGGCGCTTC |
| human MT-ATP8 | Forward | ATACTACCGTATGGCCCACC |
|  | Reverse | GGGCTTTGGTGAGGGAGGTA |
| human MT-CO2 | Forward | GCTGTCCCCACATTAGGCTT |
|  | Reverse | CGATGGGCATGAAACTGTGG |
| human MT-CO3 | Forward | ACCAATGATGGCGCGATGTA |
|  | Reverse | GGCTGGAGTGGTAAAAGGCT |
| human MT-CYB | Forward | CCCACCCCATCCAACATCTC |
|  | Reverse | GCGTCTGGTGAGTAGTGCAT |
| human MGST3 | Forward | TTTGCGTGGGGAGTAGTTGG |
|  | Reverse | AAAGTTGTTTCCTTGGCAAATGTC |
| human ACTB | Forward | CTTCGCGGGCGACGAT |
|  | Reverse | CCACATAGGAATCCTTCTGACC |
